# Sex‐ and Age‐Specific Associations Between Visceral Fat‐to‐Muscle Ratio and Bone Mineral Density in Children and Adolescents

**DOI:** 10.1155/ije/3711569

**Published:** 2026-01-06

**Authors:** Fang Jin, Pengzheng Yu, Zhongxin Zhu

**Affiliations:** ^1^ Department of Osteoporosis Care and Control, Xiaoshan Affiliated Hospital of Wenzhou Medical University, Hangzhou, 311200, Zhejiang, China

**Keywords:** adolescent, body composition, bone density, growth and development, sex characteristics

## Abstract

**Background:**

Childhood and adolescence are critical periods for skeletal development, yet the sex‐ and age‐specific relationships between body composition and bone mineral density (BMD) remain inadequately explored.

**Methods:**

This study analyzed data from 6328 participants aged 8–19 years from the NHANES (2011–2018) using dual‐energy X‐ray absorptiometry to assess visceral adipose tissue area (VATA), skeletal muscle mass (SMM), and total BMD. Multivariate linear regression models were employed to examine the associations, accounting for potential confounders.

**Results:**

Initial analyses indicated a positive correlation between VATA and BMD, which reversed after covariate adjustment. SMM consistently showed positive correlations with BMD, particularly in girls aged 8–11 years. The visceral adipose tissue to SMM ratio exhibited significant negative correlations with BMD, especially in boys aged 12–19 years.

**Conclusions:**

These findings highlight the critical importance of balanced body composition for skeletal health during development, suggesting that targeted interventions to optimize muscle mass and reduce visceral fat may enhance bone density in children and adolescents.

## 1. Introduction

Childhood and adolescence are critical developmental windows for establishing lifelong skeletal and metabolic health, with body composition serving as a key determinant of future physiological trajectories [1, 2]. During these formative years, the intricate balance between muscle mass and fat mass significantly influences bone health and the future risks of osteoporosis, fractures, and metabolic disorders [3, 4]. The transition from childhood to adolescence is marked by profound hormonal shifts that drive distinctive patterns of muscle accretion, adipose distribution, and skeletal maturation [5, 6]. Sex‐specific hormonal cascades, predominantly mediated by testosterone and estrogen, serve as fundamental molecular architects that orchestrate complex remodeling of body composition and skeletal architecture [7, 8].

Existing research has been limited by oversimplified metrics such as body mass index (BMI) or total fat, which inadequately capture the nuanced interactions between visceral adiposity and muscle dynamics [9, 10]. Visceral adipose tissue emerges as a critical mediator, secreting proinflammatory cytokines and adipokines that may disrupt bone metabolic processes [11]. Conversely, skeletal muscle mass (SMM) appears to exert a protective influence through mechanical loading and myokine‐mediated osteogenic stimulation [12]. However, the differential contributions of these factors across sex and developmental stages remain incompletely understood.

Traditional approaches predominantly rely on BMI as a crude proxy for adiposity, fundamentally failing to differentiate between visceral and subcutaneous fat or to account for variations in muscle mass. Notably, comprehensive population‐based studies employing dual‐energy X‐ray absorptiometry (DXA) to validate body composition and bone mineral density (BMD) are remarkably sparse, particularly within pediatric populations. To address these critical knowledge gaps, this study employs a substantial sample size to systematically investigate sex‐specific correlations between visceral adipose tissue area (VATA), SMM, and their ratio in relation to total BMD in children and adolescents. Drawing on existing literature that underscores the roles of visceral adiposity and skeletal muscle in bone metabolism, we hypothesized that a higher visceral adipose tissue area‐to‐skeletal muscle mass ratio (VSR) would be inversely associated with BMD in this demographic. Additionally, we anticipated that this association would exhibit significant variation by sex and age, attributable to sex‐specific hormonal fluctuations during pubertal development.

## 2. Methods

### 2.1. Study Design and Population

The National Health and Nutrition Examination Survey (NHANES) is an ongoing study coordinated by the National Center for Health Statistics, aimed at evaluating the health and nutritional status of adults and children in the United States. The survey employs a complex, multistage, probability sampling design to recruit a representative sample of the noninstitutionalized civilian population. Participants are initially contacted through household interviews, after which eligible individuals undergo comprehensive physiological assessments at mobile examination centers (MECs). NHANES was conducted in accordance with the guidelines of the Declaration of Helsinki, and all procedures involving human subjects received approval from the National Center for Health Statistics Research Ethics Review Board. Written informed consent was obtained from all participants aged 18 years and older, while parental or guardian consent was secured for minors.

**Figure 1:**
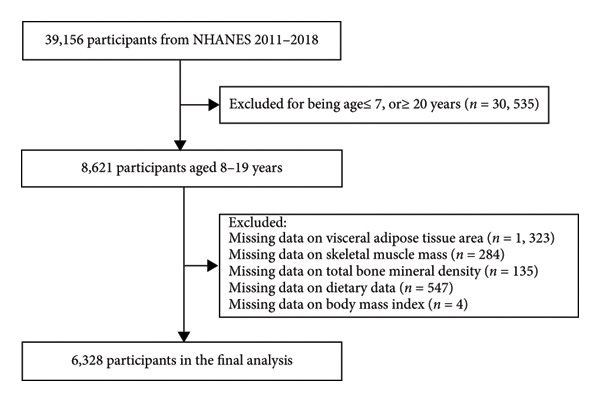
Sample inclusion and exclusion flowchart.

This cross‐sectional study utilized data amalgamated from four consecutive NHANES cycles (2011–2018). The initial population comprised 8621 children and adolescents aged 8–19 years. A sequential exclusion process was applied to individuals with missing data on VATA (*n* = 1323), SMM (*n* = 284), total BMD (*n* = 135), dietary data (*n* = 547), or BMI (*n* = 4). After applying these criteria, the final analytical sample consisted of 6328 participants with complete data for all variables of interest (Figure 1), providing a robust sample size for subsequent statistical analyses.

### 2.2. Variables

During the 2011–2018 cycles, DXA whole‐body scans were administered to eligible survey participants aged 8–59 years. VATA, representing fat within the abdominal cavity, was measured at the approximate interspace between the L4 and L5 vertebrae. SMM was calculated as the sum of lean mass in the arms and legs. All DXA assessments—including VATA, SMM, and total BMD—were conducted by trained radiologic technologists at MECs using a Hologic QDR 4500A fan‐beam densitometer, in accordance with standardized protocols. Drawing from existing literature, we identified and collected data on key potential confounders, including age (stratified into 8–11, 12–15, and 16–19 years), sex, race/ethnicity (categorized as non‐Hispanic White, non‐Hispanic Black, Mexican American, and other), family income‐to‐poverty ratio, BMI, moderate activities, and dietary intakes of protein, vitamin D, and calcium. Moderate activity was defined using a survey question on typical weekly moderate‐intensity activities of ≥ 10 min. Dietary intakes were averaged from two 24‐hour dietary recalls collected during the MEC visit and a follow‐up telephone interview.

### 2.3. Statistical Analyses

Participant characteristics were rigorously stratified by sex to comprehensively present baseline distribution. Continuous variables were expressed as mean ± standard deviation, while categorical variables were presented as percentages. Statistical comparisons employed precision‐driven approaches: *χ* ^2^ tests for categorical variables, one‐way ANOVA for normally distributed continuous variables, and Kruskal–Wallis *H* tests for skewed distributions, ensuring methodologically appropriate statistical treatment across diverse variable types.

A comprehensive multivariate linear regression framework was implemented to evaluate complex associations between VATA, SMM, VSR, and BMD. Adhering to STROBE statement recommendations [13], a hierarchical modeling strategy was developed: Model 1 (unadjusted), Model 2 (adjusted for age, sex, and race), and Model 3 (fully adjusted for all screened covariates). Subgroup analyses utilized stratified linear regression models to explore potential effect modifications. Recognizing the intricate biological relationships, advanced statistical techniques including smooth curve fitting and generalized additive models were employed to explore and confirm potential nonlinear associations.

Statistical computations were performed using R software (Version 3.4.3) and EmpowerStats (X&Y Solutions, Inc., Boston, MA), with statistical significance defined as two‐sided *p* values less than 0.05.

## 3. Results

Analysis of participant characteristics revealed significant sex‐based differences across multiple metabolic and anthropometric parameters (Table 1). Boys demonstrated significantly elevated parameters across multiple critical domains, including dietary nutrient acquisition, VATA, SMM, and BMD.

**Table 1:** The characteristics of participants according to sex.

	Boys (*n* = 3264)	Girls (*n* = 3064)	*p* value
Age (years)	13.2 ± 3.4	13.2 ± 3.4	0.885
Race/ethnicity (%)			0.065
Non‐Hispanic White	27.9	26.8	
Non‐Hispanic Black	25.0	23.4	
Mexican American	20.0	22.5	
Other race/ethnicity	27.1	27.3	
Family income‐to‐poverty ratio (%)			0.201
< 1.5	44.3	46.0	
≥ 1.5	47.4	46.7	
Unrecorded	8.3	7.3	
Moderate activities (%)			0.087
Yes	27.3	24.9	
No	25.9	27.2	
Unrecorded	46.8	47.9	
Body mass index (kg/m^2^)	22.2 ± 5.8	22.6 ± 6.1	0.005
Dietary protein intake (g/d)	81.0 ± 36.7	63.8 ± 24.5	< 0.001
Dietary vitamin D intake (μg/d)	8.4 ± 23.6	7.1 ± 13.2	0.009
Dietary calcium intake (mg/d)	1101.4 ± 556.5	899.0 ± 434.8	< 0.001
Visceral adipose tissue area ratio (cm^2^)	48.6 ± 21.5	41.1 ± 29.4	< 0.001
Skeletal muscle mass (kg)	18.6 ± 7.5	14.6 ± 4.6	< 0.001
Visceral adipose tissue area to skeletal muscle mass ratio (kg/cm^2^)	2.9 ± 1.4	2.8 ± 1.6	< 0.001
Total bone mineral density (mg/cm^2^)	959.1 ± 163.9	939.4 ± 145.0	< 0.001

Comprehensive statistical modeling across progressive quartiles unveiled intricate relationships between VATA, SMM, VSR, and BMD, systematically documented in Table 2. Initial unadjusted analysis (Model 1) demonstrated a positive association between VATA and BMD (*β* = 1.4, 95% CI: 1.2–1.5), which dramatically reversed after rigorous covariate adjustment in Model 3 (*β* = −1.6, 95% CI: −1.7 to −1.4). Notably, SMM consistently exhibited positive correlations, while the VSR demonstrated consistent negative correlations across all statistical models, with the highest quartiles revealing most pronounced associations in Model 3, substantiated by Figure 2.

**Table 2:** Correlations of visceral adipose tissue area (cm^2^), skeletal muscle mass (kg), and their ratio with total bone mineral density (mg/cm^2^).

	Model 1	Model 2	Model 3
	*β* (95% CI)	*β* (95% CI)	*β* (95% CI)
Visceral adipose tissue area	1.4 (1.2, 1.5)^∗∗∗^	0.4 (0.3, 0.5)^∗∗∗^	−1.6 (−1.7, −1.4)^∗∗∗^
Q1	Reference	Reference	Reference
Q2	52.9 (42.5, 63.4)	15.6 (8.8, 22.4)	−1.5 (−8.0, 5.0)
Q3	96.3 (85.8, 106.7)	31.7 (24.8, 38.6)	−7.6 (−14.5, −0.6)
Q4	98.7 (88.3, 109.2)	37.1 (30.3, 43.8)	−51.4 (−60.2, −42.6)
*p* for trend	< 0.001	< 0.001	< 0.001
Skeletal muscle mass	18.9 (18.6, 19.3)^∗∗∗^	12.0 (11.5, 12.4)^∗∗∗^	13.5 (12.9, 14.1)^∗∗∗^
Q1	Reference	Reference	Reference
Q2	138.9 (132.2, 145.6)	62.6 (56.5, 68.6)	56.9 (50.6, 63.2)
Q3	222.5 (215.8, 229.1)	106.1 (99.2, 113.0)	94.9 (87.2, 102.5)
Q4	334.6 (327.9, 341.3)	186.3 (178.0, 194.5)	166.1 (156.0, 176.1)
*p* for trend	< 0.001	< 0.001	< 0.001
Visceral adipose tissue area to skeletal muscle mass ratio	−36.1 (−38.5, −33.7)^∗∗∗^	−8.6 (−10.3, −7.0)^∗∗∗^	−25.2 (−26.9, −23.5)^∗∗∗^
Q1	Reference	Reference	Reference
Q2	−36.5 (−46.5, −26.4)	−27.7 (−34.0, −21.5)	−38.2 (−43.9, −32.6)
Q3	−99.7 (−109.7, −89.6)	−35.4 (−41.9, −29.0)	−65.0 (−71.0, −59.0)
Q4	−150.2 (−160.3, −140.2)	−40.8 (−47.6, −34.0)	−97.2 (−104.1, −90.3)
*p* for trend	< 0.001	< 0.001	< 0.001

*Note:* Model 1: no covariates were adjusted. Model 2: age, sex, and race were adjusted. Model 3: age, sex, race, family income‐to‐poverty ratio, moderate activities, body mass index, protein intake, vitamin D intake, and calcium intake were adjusted.

^∗^ *p* < 0.05, ^∗∗^ *p* < 0.01, and ^∗∗∗^ *p* < 0.001.

Figure 2The correlations of visceral adipose tissue area (a), skeletal muscle mass (b), and visceral adipose tissue area‐to‐skeletal muscle mass ratio (c) with total bone mineral density. Age, sex, race, family income‐to‐poverty ratio, moderate activities, body mass index, protein intake, vitamin D intake, and calcium intake were adjusted.

**Figure:**
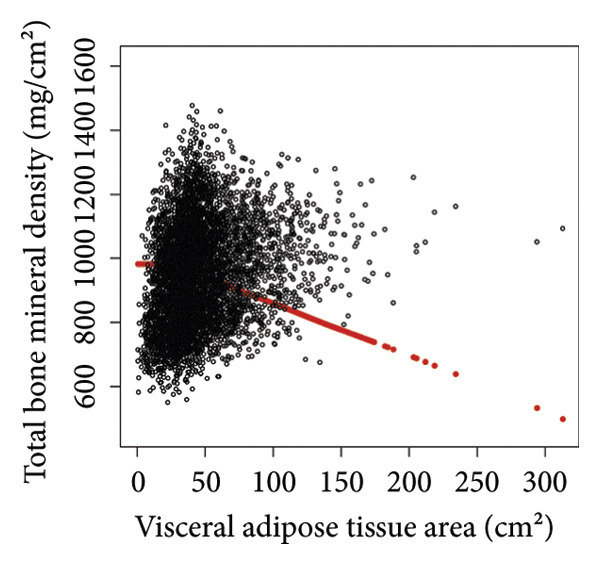
(a)

**Figure:**
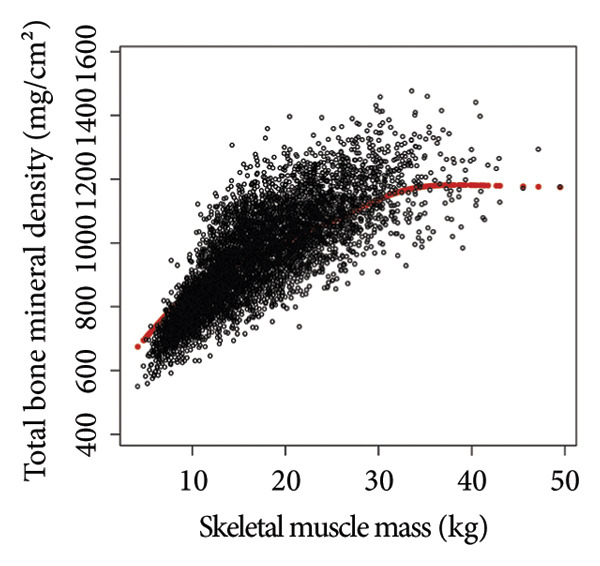
(b)

**Figure:**
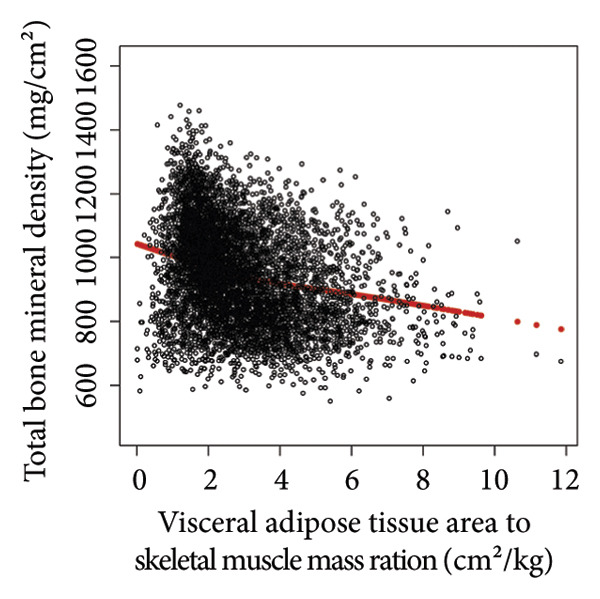
(c)

Stratified subgroup analyses across age, sex, and race/ethnicity (Figure 3) revealed nuanced developmental patterns: VATA demonstrated a negative BMD association across all subgroups, with the most significant correlation observed in boys aged 12–15 years (*β* = −2.7, 95% CI: −3.2 to −2.3). Conversely, SMM displayed positive BMD associations, peaking in girls aged 8–11 years (*β* = 25.0, 95% CI: 23.5–26.5). The VSR exhibited stronger negative BMD correlations in boys aged 12–15 years (*β* = −61.9, 95% CI: −67.1 to −56.6) and 16–19 years (*β* = −63.6, 95% CI: −72.8 to −54.3), with markedly weaker associations in girls and younger boys. Generalized additive models and smooth curve fitting validated these complex body composition–BMD interactions (Figures 4 and 5).

Figure 3Subgroup analysis of the correlations of visceral adipose tissue area (a), skeletal muscle mass (b), and visceral adipose tissue area‐to‐skeletal muscle mass ratio (c) with total bone mineral density. Age, sex, race, family income‐to‐poverty ratio, moderate activities, body mass index, protein intake, vitamin D intake, and calcium intake were adjusted. In the subgroup analysis, the model is not adjusted for the stratification variable itself. Abbreviations: SMM = skeletal muscle mass; VATA = visceral adipose tissue area; VSR = visceral adipose tissue area to skeletal muscle mass ratio.

**Figure:**
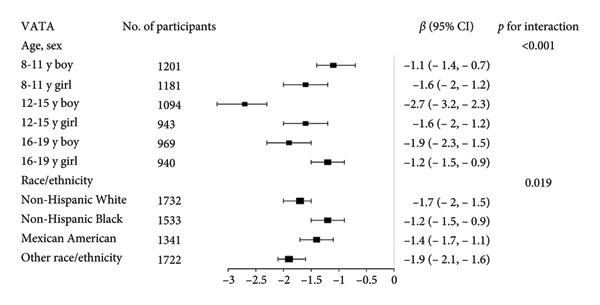
(a)

**Figure:**
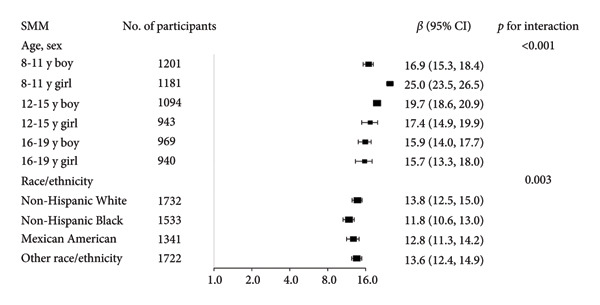
(b)

**Figure:**
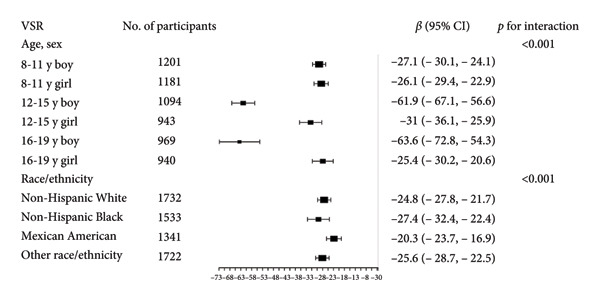
(c)

Figure 4Age‐ and sex‐stratified smooth curve analysis of the correlations of visceral adipose tissue area (a), skeletal muscle mass (b), and visceral adipose tissue area‐to‐skeletal muscle mass ratio (c) with total bone mineral density. Race, family income‐to‐poverty ratio, moderate activities, body mass index, protein intake, vitamin D intake, and calcium intake were adjusted.

**Figure:**
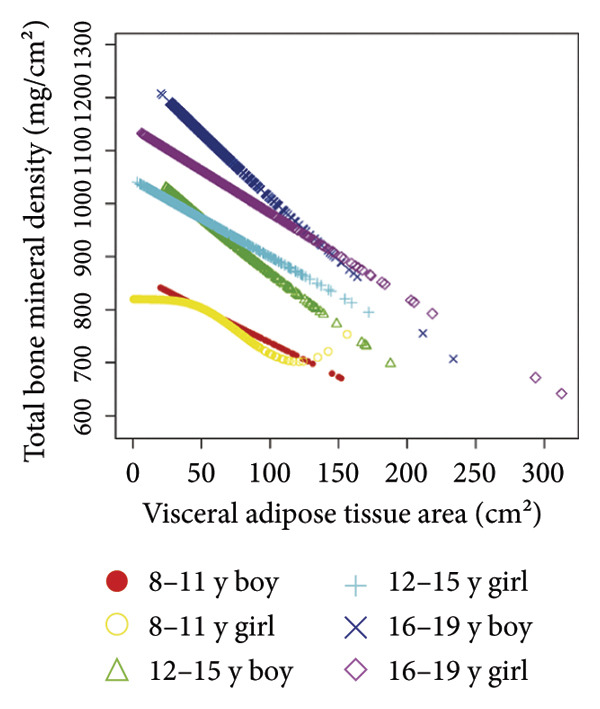
(a)

**Figure:**
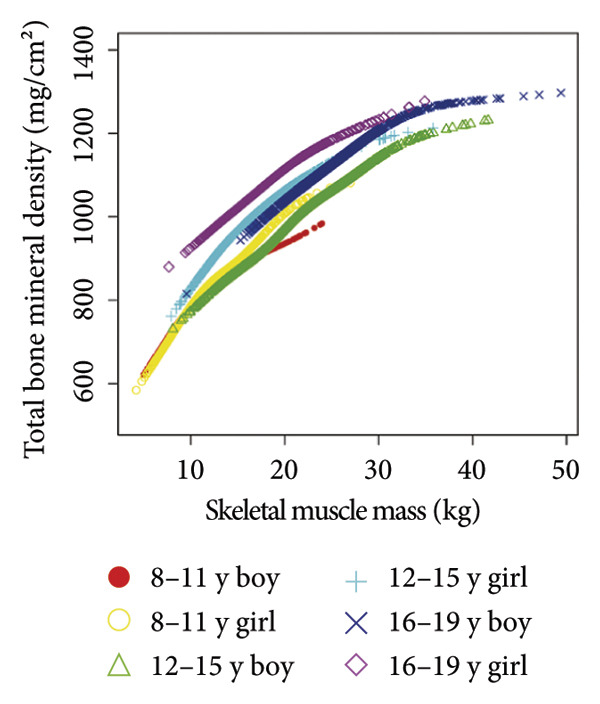
(b)

**Figure:**
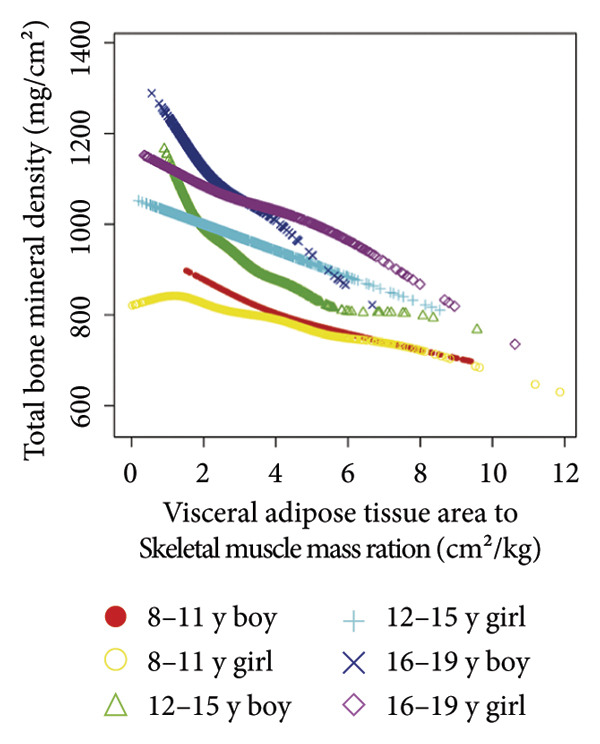
(c)

Figure 5Race‐stratified smooth curve analysis of the correlations of visceral adipose tissue area (a), skeletal muscle mass (b), and visceral adipose tissue area‐to‐skeletal muscle mass ratio (c) with total bone mineral density. Age, sex, family income‐to‐poverty ratio, moderate activities, body mass index, protein intake, vitamin D intake, and calcium intake were adjusted. In the subgroup analysis, the model is not adjusted for the stratification variable itself.

**Figure:**
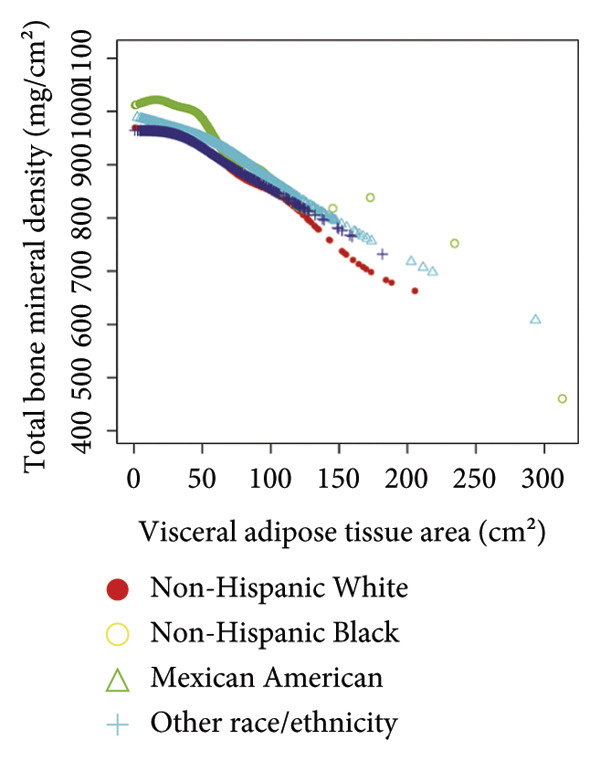
(a)

**Figure:**
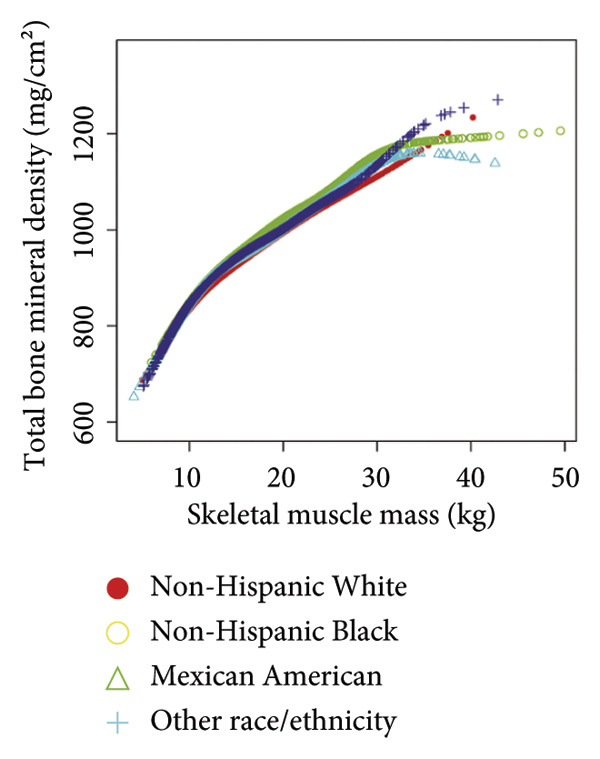
(b)

**Figure:**
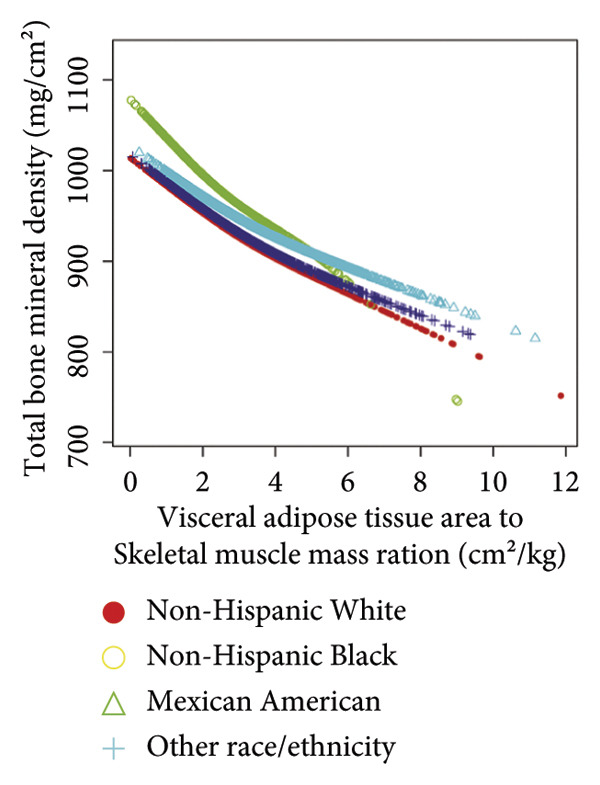
(c)

## 4. Discussion

Our study provides unprecedented insights into the intricate relationship between VSR and BMD in children and adolescents, revealing nuanced sex‐ and age‐dependent variations. The most striking finding is a pronounced negative association between VSR and BMD, particularly evident in boys aged 12–19 years, underscoring the pivotal role of body composition balance in skeletal development during critical developmental stages.

Existing evidence substantiates the crucial role of muscle mass in bone health. Skeletal muscle contributes substantially to bone development, with approximately 40% of postnatal bone growth attributed to muscle development [14]. Studies in Colombian adolescents have demonstrated that muscle mass and fitness positively influence bone health, suggesting targeted strength interventions could optimize skeletal development [15]. Conversely, adipose tissue—especially visceral fat—exhibits a predominantly negative relationship with bone density. Regional fat deposition correlates inversely with bone density, potentially disrupting critical metabolic processes [11, 16].

Sex‐specific physiological variations provide further context to these observations. Boys consistently demonstrate higher body fat percentages and greater muscle mass throughout childhood [17, 18]. This increased muscle mass contributes to enhanced bone density through mechanical stress, with body composition dynamically changing during adolescence. The increasing muscle mass and decreasing visceral fat with age likely drive improvements in bone density, particularly during adolescence when muscle development is critical for achieving peak bone mass. Hormonal dynamics during puberty mediate these compositional changes. The pronounced negative VSR–BMD association in older boys (12–19 years) reflects complex metabolic shifts characterized by rapid growth and hormonal fluctuations [19–21]. Boys typically enter puberty later than girls, with significant testosterone increases occurring between ages 11 and 17 [22], which may explain the weaker association between VSR and BMD in younger boys (8–11 years).

Traditional anthropometric metrics, such BMI, provide oversimplified assessments that fail to capture the intricate nuances of body composition [23]. In contrast, the fat‐to‐muscle ratio emerges as a more sophisticated measure, offering a refined evaluation of body fat relative to lean mass and serving as a superior indicator of health risks associated with adiposity [24, 25]. Our study leverages DXA to provide unprecedented precision in body composition analysis. To our knowledge, this represents the first comprehensive investigation utilizing such precise detection methods to elucidate the complex relationship between fat‐to‐muscle ratio and BMD in pediatric populations. The findings suggest promising therapeutic implications: Targeted interventions aimed at enhancing muscle mass and reducing visceral fat could potentially optimize bone density during critical developmental stages.

However, several methodological limitations warrant careful consideration. The cross‐sectional study design inherently constrains causal inference regarding the intricate relationships between VSR and BMD. Despite rigorous covariate adjustments, potential residual confounding remains a significant concern. Unmeasured factors, including genetic predispositions, nuanced physical activity patterns, and precise hormonal measurements, may subtly influence the observed associations. Furthermore, the reliance on single‐point measurements presents a notable constraint. This approach fails to capture the dynamic temporal variations in body composition that characterize critical developmental periods. The complex, evolving nature of pediatric physiological development necessitates more longitudinal approaches to fully comprehend these intricate biological interactions.

## 5. Conclusion

Our findings reveal a significant negative association between VSR and BMD in children and adolescents, particularly pronounced in adolescent boys. This underscores the critical importance of maintaining a balanced body composition during developmental stages. These observations have substantial implications for pediatric health interventions, suggesting that promoting physical activity and muscle development may be crucial strategies for optimizing bone health.

## Ethics Statement

The Ethics Review Board of the National Center for Health Statistics approved all NHANES protocols, and written informed consent was obtained from all participants or their parents/legal guardians.

## Consent

Please see the Ethics Statement.

## Conflicts of Interest

The authors declare no conflicts of interest.

## Author Contributions

Fang Jin and Pengzheng Yu contributed to data collection, analysis, and writing of the manuscript. Zhongxin Zhu contributed to study design, analysis, writing, and editing of the manuscript.

## Funding

This study received no funding.

## Data Availability

The data of this study are publicly available on the NHANES website (https://wwwn.cdc.gov/nchs/nhanes/).
